# Ultra-sensitive chemiluminescence imaging DNA hybridization method in the detection of mosquito-borne viruses and parasites

**DOI:** 10.1186/s13071-017-1975-1

**Published:** 2017-01-25

**Authors:** Yingjie Zhang, Qiqi Liu, Biao Zhou, Xiaobo Wang, Suhong Chen, Shengqi Wang

**Affiliations:** 1 Beijing Institute of Radiation Medicine, Beijing, 100850 People’s Republic of China; 2 Beijing Key Laboratory of New Molecular Diagnosis Technologies for Infectious Diseases, Beijing, 100850 People’s Republic of China; 3 Postdoctoral Research Workstation, 210th Hospital of the Chinese People’s Liberation Army, Dalian, 116021 People’s Republic of China

**Keywords:** Mosquito-borne viruses, Mosquito-borne parasites, Chemiluminescence, DNA hybridization

## Abstract

**Background:**

Mosquito-borne viruses (MBVs) and parasites (MBPs) are transmitted through hematophagous arthropods-mosquitoes to homoiothermous vertebrates. This study aims at developing a detection method to monitor the spread of mosquito-borne diseases to new areas and diagnose the infections caused by MBVs and MBPs.

**Methods:**

In this assay, an ultra-sensitive chemiluminescence (CL) detection method was developed and used to simultaneously detect 19 common MBVs and MBPs. In vitro transcript RNA, virus-like particles (VLPs), and plasmids were established as positive or limit of detection (LOD) reference materials.

**Results:**

MBVs and MBPs could be genotyped with high sensitivity and specificity. The cut-off values of probes were calculated. The absolute LODs of this strategy to detect serially diluted in vitro transcribed RNAs of MBVs and serially diluted plasmids of MBPs were 10^2^–10^3^copies/μl and 10^1^–10^2^copies/μl, respectively. Further, the LOD of detecting a strain of pre-quantified JEV was 10^1.8^–10^0.8^PFU/ml, fitted well in a linear regression model (coefficient of determination = 0.9678).

**Conclusions:**

Ultra-sensitive CL imaging DNA hybridization was developed and could simultaneously detect various MBVs and MBPs. The method described here has the potential to provide considerable labor savings due to its ability to screen for 19 mosquito-borne pathogens simultaneously.

**Electronic supplementary material:**

The online version of this article (doi:10.1186/s13071-017-1975-1) contains supplementary material, which is available to authorized users.

## Background

Mosquito-borne viruses (MBVs) are transmitted from hematophagous arthropod/mosquitoes to homoiothermous vertebrates. As competent hosts, arthropods can support large virus inocula, and transmit them from an infected donor to a recipient during blood-feeding. Three major families in MBVs include the *Togaviridae*, *Flaviviridae*, and *Bunyaviridae* [1]. The globally pandemic MBV species encompass dengue virus (DENV), Japanese encephalitis virus (JEV), yellow fever virus (YFV), West Nile virus (WNV) [2] and chikungunya virus (CHIKV) [3]. In China, MBVs severely affect people living in tropical regions and rural areas. There are more than 70 identified flaviviruses, of which 35 can affect humans [4]. Factors like global warming, increased population mobility, and urbanization influence the epidemic scope of MBVs and sometimes even result in sudden outbreak [5]. In 1999, the outbreak of WNV in New York resulted in mass-scale animal mortality [6]. In 2012, the outbreak of WNV in Texas resulted in 1,868 human infections (89 deaths) and an economic cost of more than $47 million [7]. In 2010, the outbreak of CHIKV in Guangdong province of China led to 173 cases of human infections [8]. From 2005 to 2006, more than 1.3 million cases of CHIKV infections were reported in India [9]. The infected cases of DENV recorded by the World Health Organization (WHO) were approximately 0.9 million [10]. However, actual infected cases worldwide are likely to be more than 390 million; 96 million infected patients show the clinical or sub-clinical symptoms of DENV infection [11]. Each year, more than 20,000 casualties are reported due to severe infections of DENV [12].

In addition, malaria parasites and lymphatic filariae, borne by mosquitoes, are currently the most common parasites in the world [13, 14]. Malaria and lymphatic filariasis have been considered as the world’s highest priority control and eradication parasitic diseases due to their wide prevalence [15, 16]. In tropical regions, these two parasites share the same propagation host and may also co-infect humans [17]. Mosquito-borne parasites (MBPs) infecting humans include *Plasmodium vivax*, *Plasmodium malariae*, *Plasmodium falciparum* and *Plasmodium ovale*. Malaria is rampant in Asia, Africa, and Latin America, with half of the world’s population living in malaria-risk areas. In 2006, 250 million people were infected by malaria and approximately 1 million deaths were reported, among which children (<5 years) and pregnant women were most affected [18]. Mosquito-borne lymphatic filariae include *Wuchereria bancrofti*, *Brugia malayi* and *Brugia timori*. Approximately 1.3 billion people are living in the lymphatic filariasis-risk areas. Following malaria, lymphatic filariasis is the second most prevalent arthropod-borne disease worldwide, which affects 128 million people each year in Asia, Africa, Western Pacific, and parts of America [19].

The spread of the mosquito-borne pathogens from the epidemic areas to new ones poses a serious threat to the monitoring, prevention, and control of disease [20]. Efficient prevention, control, and treatment of mosquito-borne diseases rely on the rapid detection of the mosquito-borne pathogens and accurate diagnosis. Currently, methods for detecting MBVs and MBPs are mainly based on genetic assays, which include real-time PCR [21–25], isothermal amplification [26], oligonucleotide microarray [27] and sequencing [28]. However, these methods were designed for detecting only certain species of MBVs or MBPs. A method that can simultaneously detect common MBVs and MBPs has not yet been reported. In this assay, a reliable and portable, ultra-sensitive chemiluminescence (CL) imaging DNA hybridization was developed and used to simultaneously detect common MBVs and MBPs. In vitro transcript RNA, virus-like particles, and plasmids were established as positive or limit of detection (LOD) reference materials. The specificity and sensitivity of the method was validated in cultivated MBVs, simulated mosquito samples, reference materials, and non-MBVs.

## Methods

### Specimen collection and processing

Cultivated MBVs strains were obtained from the Institute of Microbiology Epidemiology of the Academy of Military Medical Sciences and National Institute for Viral Disease Control and Prevention, China CDC. Lysates (TIANamp Virus RNA Kit, Tiangen Biotech Beijing Co., Ltd.) were added to all the viral samples prior to experiments. Total RNAs were extracted using TIANamp Virus RNA Kit as described in the manufacturer’s protocol (Tiangen Biotech Beijing, China) and stored at -70 °C until use. Uninfected *Aedes vigilax* were obtained from the Institute of Microbiology Epidemiology of Academy of Military Medical Sciences and stored at -70 °C until use.

### Primers and probes design and evaluation

In this assay, 12 MBVs and 7 MBPs were chosen for detection: DENV1-4, JEV, YFV, WNV, St. Louis encephalitis virus (SLEV), CHIKV, eastern equine encephalitis virus (EEEV), western equine encephalitis virus (WEEV), Venezuelan equine encephalitis virus (VEEV), *P. vivax*, *P. malariae*, *P. falciparum*, *P. ovale*, *W. bancrofti*, *B. malayi* and *B. timori*. Complete genomic sequences of these MBVs, 18S rRNA gene sequences of malaria parasites, SspI repeat DNA sequence of *W. bancrofti*, and Hhal repeat region sequences of *B. malayi* and *B. timori* were downloaded from the GenBank database. The sequences were aligned using AlignX (a component of the Vector NTI Advance 10.3.0) to compare the homology between potential targets belong to the same genus. Gene-specific primers (GSPs) were derived from published literature or designed from conserved regions using Primer Premier 5 (PREMIER Biosoft International, Palo Alto, USA). Reverse primers used in Multiplex RT-PCR or PCR were biotin-labelled at 5′ end to form a CL reaction. The capture probes, which were used to capture the target DNA amplification fragments, were designed by Oligo 7 (Molecular Biology Insights, Inc., Colorado Springs, USA). Continuous homologous sequences of congeners were excluded. A human-original sequence was used as negative control probe. A repeat sequence of (T)_12_ with an amino-labeled 3′-end was connected to the 3′-end of all the probes to fix the repeat sequence with the aldehyde-chip surface. A repeat sequence of (T)_20_ with a biotin-labeled 5′-end and an amino-labeled 3′-end served as a quality control (QC) probe. All the primers and probes were confirmed by NCBI BLAST and synthesized by Sangon Biotech Co., Ltd. (Shanghai, China).

To evaluate the efficiency of primers, serially diluted viral RNAs or plasmid DNAs were amplified by RT-PCR or PCR and resolved by 2% agarose gel electrophoresis. Furthermore, the most efficient pairs of primers were biotin labeled in reverse primers, and RT-PCR or PCR was used to evaluate the DNA hybridization efficiency of the capture probes.

### Preparation of in vitro transcript RNA

To verify the LOD of this strategy and calibrate the concentration of virus-like particles (VLPs), in vitro transcribed RNAs of these MBVs were prepared. First, plasmids were constructed by cloning target gene fragments to PGM-T vector as described in the manufacturer’s protocol (Tiangen Biotech Beijing Co., Ltd.). Following digestion with FastDigest *Sal* I restriction endonuclease (ThermoFisher, Waltham, USA), the plasmids were purified by agarose gel and retrieved. Subsequently, in vitro transcribed RNAs were prepared with TranscriptAid T7 High Yield Transcription Kit (ThermoFisher), digested by deoxyribonuclease (DNase) I, and purified by phenol-chloroform extraction method as described in the manufacturer’s protocol (ThermoFisher). Further, in vitro transcribed RNAs were purified and digested by DNase I using RNeasy Mini Kit (QIAGEN, Mississauga, Canada) as described in the manufacturer’s protocol. The purified RNAs were mixed with 2 × RNA Loading Dye Solution (ThermoFisher) and resolved by 2% agarose gel electrophoresis. Finally, in vitro transcribed RNAs were quantified by UV spectrophotometry and subject to serial dilutions at a magnitude of 10 and stored at -70 °C until use.

### Preparation of virus-like particles

The maturation protein gene and coat protein gene of MS2 bacteriophage were amplified by MS2-*Bam*H I and MS2-*Hin*d III primers (modified from reference literature) [29], and the sequence was as follows: MS2-*Bam*H I, 5′-AAC *GGA TCC* CAT GGC TAT CGC TGT AGG TAG CC-3′, MS2-*Hin*d III, 5′-CAT *AAG CTT* CTT CGA CAT GGG TAA TCC TCA TGT T-3′. The bases in italics represent endonuclease sites of *Bam*H I and *Hin*d III. The PET-MS2 vector was constructed using the MS2 amplified fragment and PET-28a (+) vector (Novagen, Merck KGaA, Darmstadt, Germany) and ligated using T4 DNA ligase (New England BioLabs® Inc., Ipswich, USA) after digestion with restriction enzymes (*Bam*H I and *Hin*d III, ThermoFisher), according to the manufacturer’s protocol. The *Hin*d III restriction enzyme site and protection bases were added to the 5′ end of MBVs’ GSPs (both forward and reverse), and target genes of these mosquito-borne viruses were amplified and digested by *Hin*d III restriction enzyme. PET-MS2 vectors were also digested by *Hin*d III and dephosphorylated by alkaline phosphatase (New England BioLabs® Inc.). Target gene fragments of MBVs and PET-MS2 vectors were ligated using T4 DNA ligase and cloned to BL21 (DE3) competent cells (Tiangen Biotech Beijing Co., Ltd.), according to the manufacturer’s instructions. These bacterial plasmids were induced by isopropyl-1-thio-β-galactopyranoside (IPTG) to express VLPs and lysed by sonication. The VLPs were precipitated by PEG8000 and purified by 15–45% (w/v) sucrose density gradient ultracentrifugation at 32,000× *rpm* for 6 h/4 °C (Beckman, MAL-80 ROTOR, Brea, USA). The aliquot containing VLPs was dialyzed against PBS at 4 °C for 24 h to remove sucrose. The purified VLPs were used as positive RNA references materials.

### Multiplex asymmetric RT-PCR and PCR amplification

The GSPs of MBVs and MBPs were used in two multiplex asymmetric RT-PCR systems and one multiplex asymmetric PCR system, respectively. For optimal amplification efficiency, orthogonal experiment was used to optimize the concentration of the GSPs. Briefly, the concentration ratio of the forward and reverse primers were set to 1:5. The high, medium, and low concentrations of primers were set based on the results of preliminary experiments. The selected primer concentrations were determined by evaluation of the amplification efficiencies of the target genes. Each RT-PCR was performed in a 25 μl reaction volume, containing 12.5 μl of 2× One Step Buffer, 1.0 μl of PrimeScript One-step Enzyme Mix (DRR055A, Takara Biotechnology (Dalian) Co., Ltd., Dalian, China), 5 μl of total RNA template, and specific primer mix. RT-PCR system A amplified the target genes of mosquito-borne flavivirus included DENV, JEV, YFV, SLEV, and WNV. System A contained GSPs of DC10418 (0.2 μmol), JEV-3UTR-F2 (0.28 μmol), YFV-3UTR-F1 (0.4 μmol), WNV-3UTR-F2 (0.2 μmol), SLE-3UTR-F1 (0.1 μmol), and CDC10564 (2 μmol). RT-PCR system B amplified the target genes of mosquito-borne alphavirus included EEEV, WEEV, VEEV, and CHIKV. System B contained GSPs of EEE-E1-F (0.4 μmol), EEE-E1-R (2 μmol), CHIKV-F1 (0.2 μmol), CHIKV-R2 (1 μmol), WEE-F (0.08 μmol), WEE-R (0.4 μmol), VEEV-cap-F3 (0.12 μmol), and VEEV-cap-R3 (0.6 μmol). PCR was performed in a 25 μl reaction volume containing 2.5 μl of 10× EX Taq Buffer, 0.5 μl of TaKaRa Ex Taq HS (RR006, Takara Biotechnology (Dalian) Co., Ltd.), 200 μmol of each dNTP, 5 μl of total DNA template, and specific primer mix. PCR system amplified target genes of plasmodium and lymphatic filariae. PCR system contained GSPs of MAL-F1 (0.16 μmol), MAL-R1 (0.8 μmol), BM/WBF (0.08 μmol), WBR (0.4 μM), MGB-HhaI-For (0.16 μmol), and MGB-HhaI-Rev (0.8 μmol). Amplifications were performed on a Veriti 96-Well Thermal Cycler PCR system (Applied Biosystems, Foster City, USA). RT-PCR was performed under the following conditions: 30 min at 50 °C; 2 min at 94 °C; 45 cycles of 20 s at 94 °C, 20 s at 55 °C, and 20 s at 72 °C; and a final extension of 5 min at 72 °C. PCR was performed under the following conditions: 2 min at 95 °C; 45 cycles of 20 s at 94 °C, 20 s at 55 °C, and 20 s at 72 °C; and a final extension of 5 min at 72 °C.

### DNA Hybridization and signal detection

Captured probes of MBVs and MBPs (50 μmol) were used to establish the MBVs and MBPs capture-chip, and the spotting was repeated thrice in the vertical direction on a aldehyde modified glass slide (Baio Technology Shanghai Co., Ltd., Shanghai, China) surface with uniform proportional printing buffer (5% glycerol, 0.1% sodium dodecyl sulfate (SDS), 6× saline-sodium citrate buffer (SSC), and 2% (wt/vol) Ficoll 400) as described in previous studies [30]. The QC probe, used at 12.5 μmol final concentration, was spotted and repeated six to seven times in the horizontal direction to calibrate the CL signal values. Each aldehyde slide was divided into 10 blocks (11 × 11 mm) by a waterproof film to detect 10 different samples.

After amplification, the two multiplex RT-PCR reaction products, amplified using the same template, were blended. After 5 min of denaturation at 95 °C, the 5 μl of RT-PCR mixtures or PCR products were immediately placed on ice for 5 min and mixed with 5 μl of DNA hybridization buffer (8× SSC, 0.6% SDS, 10% formylamine, and 10× Denhardt). DNA hybridization mixtures (10 μl) were hybridized on the MBVs or MBPs capture-chip for 1 h at 45 °C. The capture-chip was washed for 30 s each with 1× SSC and 0.2% SDS, 0.2× SSC, and 0.1× SSC at room temperature. The capture-chip was incubated with 15 μl of streptavidin-horseradish peroxidase (Str-HRP, Sigma-Aldrich, St. Louis, USA) for 25 min at 37 °C. Subsequently, the capture-chip was washed with PBST (phosphate buffer, 0.05% Tween 20) 10 s at room temperature. Finally, 10 μl of pre-mixed CL HRP substrate luminal solution and H_2_O_2_ (Millipore Corporation, Boston, USA) were added to the capture-chip and detected immediately with a micro-light level imaging system (developed in-house, patent number CN201330010609) (The working principle of this CL imaging DNA hybridization method were showed in Fig. 2c).

### Determination of cut-off values of capture probes

In this assay, low background CL imaging and self-programming software were employed as the imaging method and the interpretation software was used to read the grey values of the probes. The grey values recorded by the software were set to be in the range of 0–256. The CL signal values of probes = mean of signal values of probes - signal values of background. The CL signal value was calibrated as follows: the calibrated CL signal value of a probe = mean of CL signal value of the probe / mean of CL signal value of QC probe (the same detection block) × 100. The cut-off values of probes were calculated as follows: cut-off value of a probe = average signal values of to detect other pathogens (except the pathogen detected by this probe) + 3 × standard deviation. Probe signal was considered positive when the value was greater than the cut-off value of the probe.

### Specificity and sensitivity

Due to the lack of MBVs and MBPs samples, the specificity of the strategy was evaluated only by 6 cultivated MBVs strains and an inactivated JEV Vaccine (Table 1). The other MBVs and MBPs were evaluated by artificial VLPs, in vitro transcribed RNAs, or plasmids. Further, a panel of non-MBVs, which include tick-borne encephalitis virus (TBEV), avian influenza A (H5N1), 2009 influenza A (H1N1) (PH1N1), seasonal influenza A (H3N2), seasonal influenza A (H1N1), Hepatitis B virus (HBV), Hepatitis C virus (HCV), adenovirus AD2, AD3, AD40, rubella virus, mumps virus, and respiratory syncytial viruses (RSV) HK6 and B, were also used to determine the specificity of the strategy.

**Table 1:** Actual virus samples used in this assay

No.	Genus	Virus	Type	Source
1	Flavivirus	Dengue virus 2	Cultivated strain	Institute of Microbiology Epidemiology of Academy of Military Medical Sciences, China
2		Japanese encephalitis virus	Cultivated strain	National Institute for Viral Disease Control and Prevention, China CDC
3		Japanese encephalitis virus	Inactivated vaccine strain	JEV Vaccine (P3)
4		Yellow fever virus	Cultivated strain	Institute of Microbiology Epidemiology of the Academy of Military Medical Sciences, China
5	Alphavirus	Eastern equine encephalitis virus	Cultivated strain	Institute of Microbiology Epidemiology of the Academy of Military Medical Sciences, China
6		Western equine encephalitis virus	Cultivated strain	Institute of Microbiology Epidemiology of the Academy of Military Medical Sciences, China
7		Chikungunya virus	Cultivated strain	Institute of Microbiology Epidemiology of the Academy of Military Medical Sciences, China

To evaluate the LOD of this strategy, in vitro transcribed RNAs or plasmids were quantified using UV spectrophotometer, and the copy numbers were calculated, serially diluted to 10-fold, and were detected. Purified VLPs were also diluted and detected to determine the relative concentrations as compared to in vitro transcribed RNA.

### Simulated detection of mosquito samples

To test simulated mosquito samples, uninfected mosquito pools of 50 *Aedes vigilax* were spiked with 50 μl 10^6^ copies/μl part of single or mixed VLPs or plasmids. Then the spiked mosquito pools were homogenized in 500 μl diluent containing 20% fetal bovine serum in phosphate-buffered saline using T10 basic ULTRA-TURRAX® small dispersing instrument (IKA, Staufen, Germany). The homogenized samples were centrifuged for 5 min at 12,000× *rpm*. Supernatants were removed to clean microfuge tubes. RNA or DNA was extracted from the supernatant using TIANamp Virus RNA Kit (Tiangen Biotech Beijing Co., Ltd.) or TIANamp Genomic DNA Kit (Tiangen Biotech Beijing Co., Ltd.), respectively, as described in the manufacturer’s protocol. Subsequently, the extracted RNAs or DNAs were amplified by RT-PCR or PCR systems and detected by the CL imaging DNA hybridization assay.

## Results

### Primers and probes design and evaluation

The amplification efficiency of 81 GSPs, obtained from published literatures, and 82 GSPs designed for this assay were precisely evaluated by serially diluted viral RNAs or plasmid DNAs. Biotin-labeled reverse primers were used for multiplex asymmetric RT-PCR or PCR to evaluate DNA hybridization efficiency of 223 capture probes. Finally, 20 GSPs and 21 probes were chosen to amplify the MBVs and MBPs targeted genes and capture the target DNA amplification fragments (Tables 2 and 3).

**Table 2:** The sequences of GSPs for multiplex asymmetric RT-PCR and PCR amplification

Pathogen	Primer	Sequence (5′ to 3′)	Location	Reference^a^	Primer source
Dengue virus	DC10418	TTGAGTAAACYRTGCTGCCTGTAGCTC	10,392–10,481	GQ398257	[21]
JEV	JEV-3UTR–F2	GGACTGGGTTAACAAATCTG	10,525–10,544	EF107523	Present study
YFV	YEF-3UTR-F1	AAACTACGGATGGAGAACCG	10,489–10,508	U54798	Present study
WNV	WNV-3UTR-F2	AAGTTGAGTAGACGGTGCTGC	10,533–10,553	EU249803	Present study
SLEV	SLE-3UTR-F1	GGATGTCAGGTAAACGGTGCT	10,451–10,471	FJ753286	Present study
Flavivirus	CDC10564^b^	GGGTCTCCTCTAACCTCTAGTCCT	10,569–10,592	GQ398257	[21]
EEEV	EEE-E1-F	ACACTAAATTCACCCTAGTTCGAT	11,376–11,399	AY705241	[22]
	EEE-1-R^b^	GTGTATAAAATTACTTAGGAGCAGCATTATG	11,492–11,522	AY705241	
CHIKV	CHIKV-F1	TGGATGAACATGGAAGTGAA	7,121–7,140	DQ443544	Present study
	CHIKV-R2^b^	GCTGTAGTGCGTACCTATTT	7,496–7,515	DQ443544	Present study
WEEV	WEE-F	AGGGATACCCCCGAAGGTT	8,220–8,238	AF214040	[23]
	WEE-R^b^	GTGAATAGCACACGGGTGGTT	8,302–8,322	AF214040	
VEEV	VEEV-cap-F3	GGGCGGCGCATGAGAGAAGC	3–22	U34999	Present study
	VEEV-cap-R3^b^	GTGACCTGCTTGGCTTCTACCTC	119–141	U34999	Present study
*Plasmodium*	MAL-F1	GGGAGTGAAGACGATCAGATACC	443–465	HQ283215	Present study
	MAL-R1^b^	CCGTGTTGAGTCAAATTAAGCC	668–689	HQ283215	Present study
*W. bancrofti*	BM/WBF	AGCGTGATGGCATCAAAGTAG	1–21	L20344	[24]
	WBR^b^	AGGTTATACCAAGCAAACAAAAA	110–132	L20344	
*B. malayi*/ *B. timori*	MGB-HhaI-For	GCAATATACGACCAGCAC	254–271	M12691	[25]
	MGB-HhaI-Rev^b^	ACATTAGACAAGGAAATTGGTT	140–161	M12691	

^a^GenBank accession numbers

^b^These primers were labeled by biotin to give off CL signals

**Table 3:** Capture probe sequences for DNA hybridization

Pathogen	Capture probe	Sequence (5′ to 3′)^a^	Location	Reference^b^
DENV	DEN-10731^c^	GGGAGGCCATGCGCCATGGAAGCTGTACGCATGGTGTAGCAGACT	10,443–10,487	GQ398257
DENV-1	DEN1-X10830	GCCCAACACCAGGGGAAG	10,520–10,537	FJ687426
DENV-2	DEN2-X10831	GCCCAAGGYGAGATGAAGCT	10,535–10,554	GQ398257
DENV-3	DEN3-X10829	GGCCCGAGCACTGAGGGAAGC	10,507–10,527	FJ898444
DENV-4	DEN4-X10714	CGTAATAATCCCTAGGGAGGCC	10,380–10,401	EF457906
JEV	JEV-10726	CCCACGGCCCAAGCCTCGTCTAGGAT	10,703–10,728	EF107523
YFV	YFV-10679	CCTCCGCTACCACCCTCCCAC	10,667–10,687	U54798
WNV	WNV-10768	AACTTCAAAGCCCAATGTCAGACCACG	10,650–10,676	EU249803
SLEV	SLE-10553	GTGCAAAGCCCCTCATTCCGACTCG	10,522–10,546	FJ753286
EEEV	EEEV-11424	GTTCGATGTACTTCCGAGCTATGGTGACGGTGG	11,393–11,425	AY705241
CHIKV	CHIKV-7427	ATGGCCACCTTTGCAAGCTC	7,421–7,440	DQ443544
WEEV	WEEV-8304	TCGAATGTCACGTTCCCATGCGACAAAC	8,277–8,304	AF214040
VEEV	VEEV-50	AAATGGAGAAAGTTCACGTTGACATCGAGG	42–71	U34999
*Plasmodium*	MAL-1207^d^	TTAGATTGCTTCCTTCAGTACCTTATGAGA	547–576	HQ283215
*P. falciparum*1	PF1-1187	CTTTCGAGGTGACTTTTAGA	532–551	HQ283215
*P. falciparum*2	PF2-1182	AGCATTTCTTAGGGAATGTTGATTTTATAT	573–602	HQ283222
*P. vivax*	PV-1173	AGAGTTTTCTCTTCGGAGTTTA	525–546	HQ283223
*P. malariae*	PM-1181	ATTCATATTAATGAGTGTTTCTTTTAGATAGCT	569–601	AF145336
*P. ovale*	PO-1152	TGGATGAAAARTTTTTAAATAAGAAAATTCCTTTC	391–425	JX977167
*W. bancrofti*	WB-53	AGTATGAATGGAATTTTTAGCAATTTTTTTG	52–82	L20344
*B. malayi*/ *B. timori*	BM-113	ATAAGCTTTTTTTAGTAGTTTTGGCACTTAATT	184–216	M12691
Quality control^e^	QC	TTTTTTTTTTTTTTTTTTTT		
Negative control^f^	NC	CAGAGATACATTGACC	1,215–1,230	NM_001128925

^a^A repeat sequence of (T)_12_ with an amino-labeled 3′-end was conjugated to the 3′-end of all the capture probes

^b^GenBank accession numbers

^c^This capture probe was used to detect all the DENVs

^d^This capture probe was used to detect all the malarial parasites

^e^A repeat sequence of (T)_20_ with an amino-labeled 3′-end and biotin-labeled 5′-end were used to calibrate the CL signal values during DNA hybridization

^f^A human-original sequence was used as negative control

### Construction of in vitro transcribed RNAs and VLPs

**Fig. 1:**
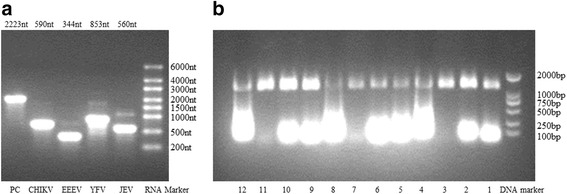
Determination of in vitro transcribed RNAs and VLPs by agarose gel electrophoresis. **a** Agarose gel electrophoresis of in vitro transcribed RNAs. The size of the in vitro transcribed RNAs were marked above each lane and compared with a RNA marker (Thermo Fisher). **b** RNase A and DNase I digestion of VLPs. Lane 1: PET-MS2 (RNase A); Lane 2: PET-MS2 (DNase I); Lane 3: PET-MS2 (RNase A + DNase I); Lane 4: PET-MS2; Lane 5: YFV VLP(RNase A); Lane 6: YFV VLP (DNase I); Lane 7: YFV VLP (RNase A + DNase I); Lane 8: YFV VLP; Lane 9: EEEV VLP (RNase A); Lane 10: EEEV VLP (DNase I); Lane 11: EEEV VLP (RNase A + DNase I); Lane 12: EEEV VLP. The size of VLPs are compared to a DNA marker (TaKaRa). The nucleic acids between 1,000–2,000 bp are resistant to both RNase A and DNase I due to their packaging in the internal section of the VLPs

Nine in vitro transcribed RNAs of MBVs were prepared to determine the LOD of the MBVs detection assay and calibrate the concentration of VLPs. After purification, in vitro transcribed RNAs were verified by agarose gel electrophoresis (Fig. 1a) and quantified by UV spectrophotometry. The copy numbers of these in vitro transcribed RNAs were calculated as: copy numbers (copies/μl) = RNA concentration (ng/μl) × Avogadro constant × 10^-9^/ (RNA length × 340 g/mol) (Table 4). The in vitro transcribed RNAs were serial diluted to 10-fold and stored at -70 °C until use.

**Table 4:** The copy numbers of in vitro transcribed RNAs quantified by UV spectrophotometer

Virus	RNA concentration (ng/μl)^a^	Length (nt)^b^	Copy number (copies/μl) ^c^	Location	Reference^d^
SLEV	61	680	2 × 10^11^	10,451–10,933	FJ753286
WNV	550	654	1 × 10^12^	10,521–10,977	EU249803
VEEV	275	339	1 × 10^12^	1–142	U34999
JEV	1810	560	5 × 10^12^	10,525–10,887	EF107523
YFV	1930	853	4 × 10^12^	10,109–10,764	U54798
EEEV	1290	344	6 × 10^12^	11,376–11,522	AY705241
CHIKV	1700	590	5 × 10^12^	7,123–7,515	DQ443544
WEEV	510	303	4 × 10^12^	8,217–8,322	AF214040
DENV2	275	395	1 × 10^12^	10,392–10,589	GQ398257

^a^RNA concentrations were quantified by UV spectrophotometer

^b^The lengths of in vitro transcribed RNAs were calculated on the length of the inserted gene fragments plus the transcript length of digested PGM-T vector

^c^Copy numbers (copies/μl) = RNA concentration (ng/μl) × Avogadro constant × 10^-9^/ (RNA length × 340 g/mol)

^d^GenBank accession numbers

**Fig. 2:**
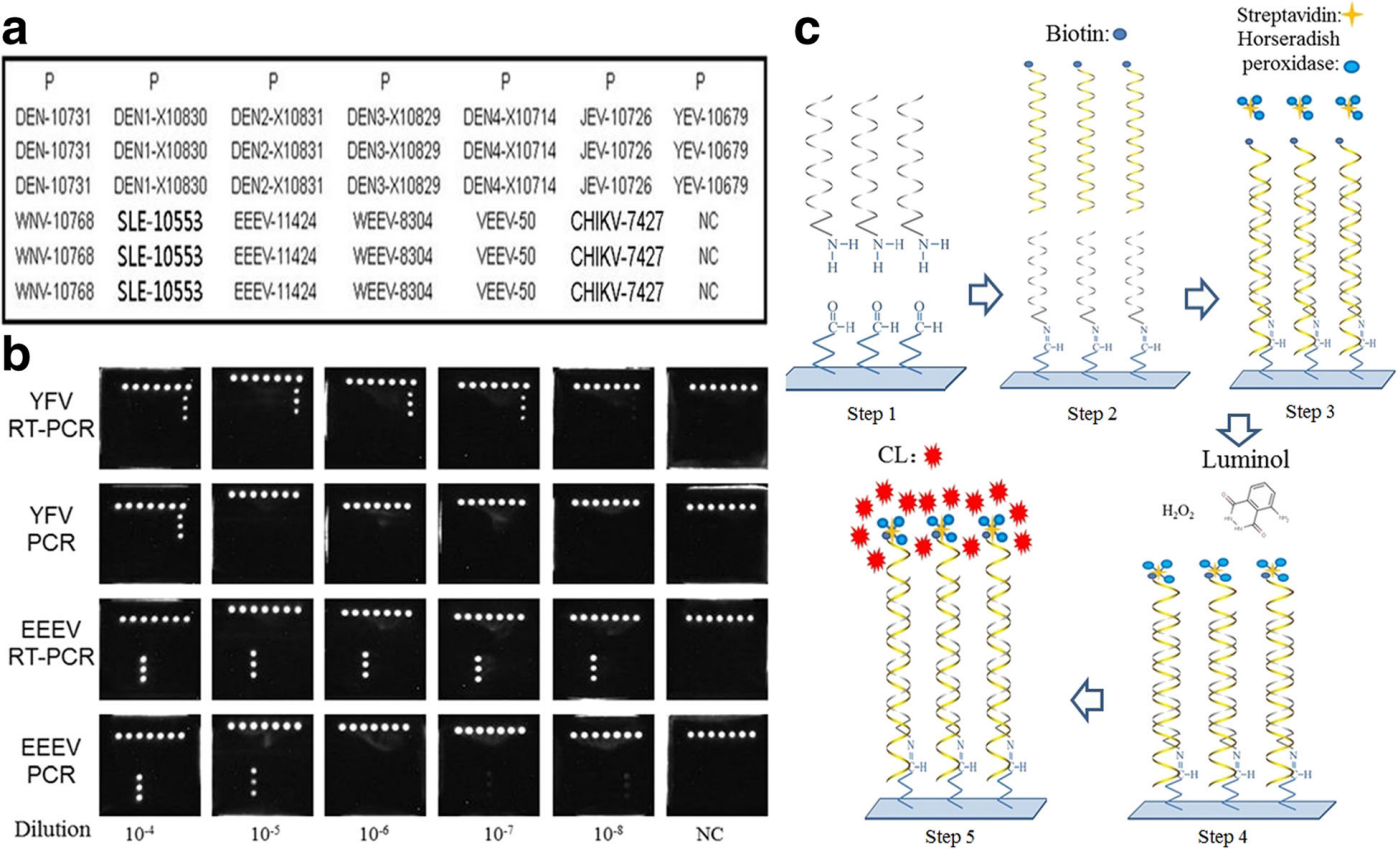
Amplification of serially diluted VLPs by both RT-PCR and PCR. **a** The layout of the MBVs hybridization capture-chip. The probe P is the QC probe. The probe NC is the negative control probe. **b** Amplifications of serially diluted VLPs by both RT-PCR and PCR (both based on the GSPs of RT-PCR system A or B). The results showed that although DNase I did not completely digest DNA templates in high concentration VLPs, the extraction of low-concentration VLPs were only detected by RT-PCR and not by PCR, demonstrating that DNA templates had been diluted to sufficiently. **c** Principle of the CL imaging DNA hybridization method. Step 1 shows that capture probes are fixed to the aldehyde-chip surface. Step 2 shows that the denatured RT-PCR products are hybridized on the capture-chip. Steps 3–5 show the principle of CL detection. Biotin is incorporated into reverse strand in RT-PCR amplification. When HRP modified streptavidin is bound, CL signal becomes illuminant by the catalyzed substrates

Twelve VLPs containing the amplified target RNAs were also prepared as the positive RNA references of the MBVs detection strategy. RNase A and DNase I digestion on the prepared VLPs showed that the nucleic acid bands between 1,000 and 2,000 bp tolerated both enzymes due to their packaging in the internal section of VLPs (Fig. 1b). To determine whether the target RNAs had been packaged in the internal regions, RNase A and DNase I digested VLPs were serially diluted (10-fold), total RNA was extracted, amplified by both multiplex RT-PCR and PCR (both based on the GSPs of RT-PCR system A or B), and detected by MBVs hybridization capture-chip. The results showed that DNase I did not completely digested the DNA templates from high concentration VLPs; the extractions from low-concentration VLPs could only be detected by RT-PCR but not PCR (Fig. 2b), thereby demonstrating that the DNA templates had been diluted to sufficiently low concentrations that cannot be detected.

### Determination of cut-off value of capture probes

In this assay, the cut-off values of a probe were calculated as follows, cut-off = average signal value to detect other pathogens (except the particular pathogen detected by this probe) + 3 × standard deviation. The cut-off values of all the probes are shown in Table 5.

**Table 5:** Cut-off values of the capture probes

Probe	Average of negative sample (grey value)	SD (grey value)	Cut-off value^a^ (grey value)
DEN-10731	0.50	0.67	2.51
DEN1-X10830	0.18	0.33	1.17
DEN2-X10831	0.85	1.55	5.50
DEN3-X10829	0.38	0.29	1.25
DEN4-X10714	0.15	0.22	0.81
JEV-10726	0.42	0.30	1.32
YFV-10679	1.90	3.80	13.30
WNV-10768	0.30	0.39	1.47
SLE-10553	0.19	0.14	0.61
EEEV-11424	0.29	0.43	1.58
WEEV-8304	0.64	0.85	3.19
VEEV-50	1.02	2.03	7.11
CHIKV-7427	0.23	0.37	1.34
MAL-1207	0.50	0.20	1.10
PF1-1187	0.10	0.14	0.52
PF2-1182	0.32	0.20	0.92
PV-1173	0.25	0.11	0.58
PM-1181	0.20	0.14	0.62
PO-1152	0.20	0.14	0.62
BM-113	0.43	0.42	1.69
WB-53	0.20	0.14	0.62

*Abbreviation*: *SD* standard deviation

^a^Cut-off value of a probe (grey value) = average signal values of to detect other pathogens (except the particular pathogen detected by this probe) + 3 × standard deviation. A probe signal was considered positive when the value was greater than the cut-off value of a given probe

### Evaluation of specificity

**Fig. 3:**
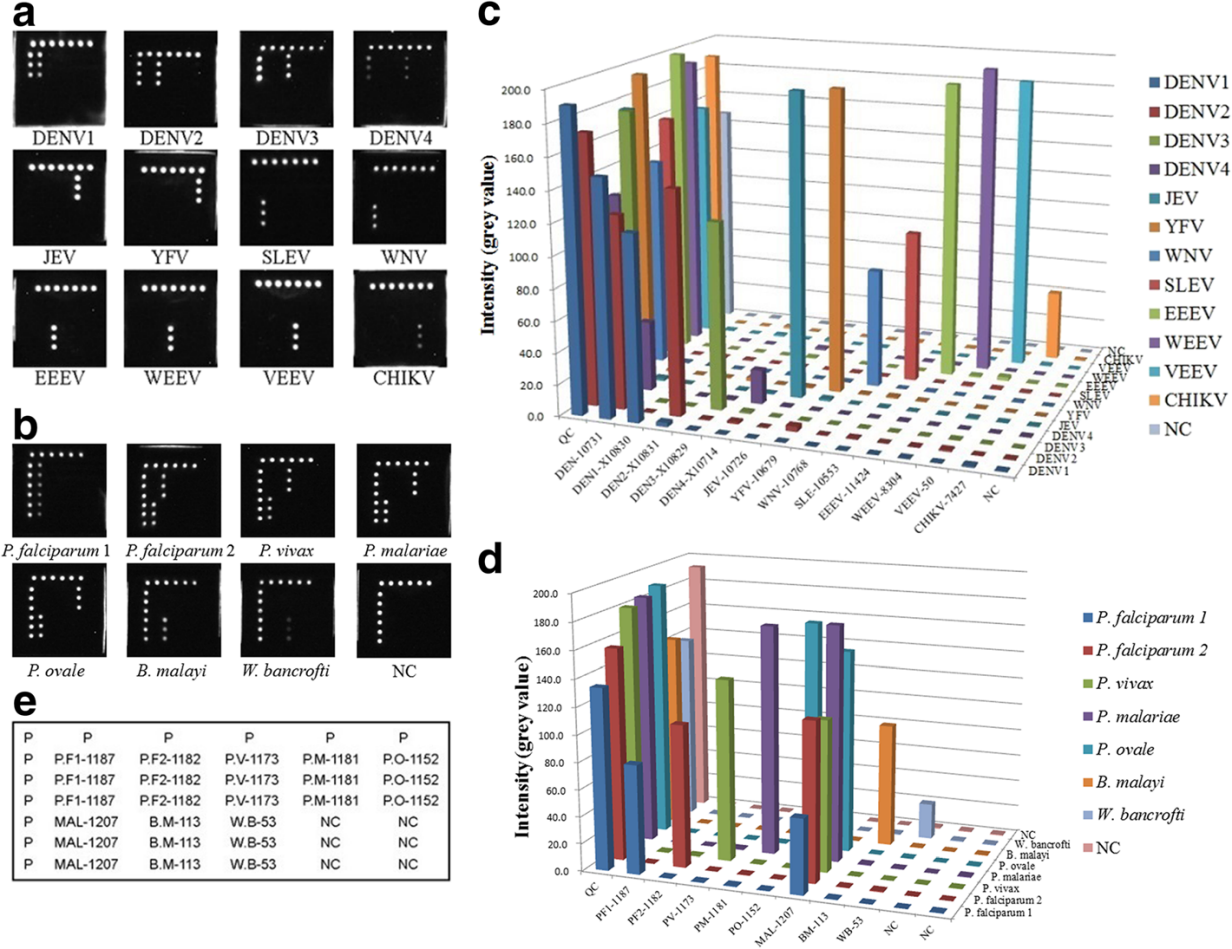
Specificity of the CL imaging DNA hybridization method. **a** and **b** Specificity of MBV and MBP detection, respectively. Cross-reaction is not present in samples and reference materials. **c** CL signal value (not calibrated) of each probe for MBVs is shown in the 3-D bar chart. **d** CL signal value (not calibrated) of each probe for the MBPs is shown in the 3-D bar chart. **e** Layout of the MBPs hybridization capture-chip. The probe P is the QC probe. The probe NC is the negative control probe

The specificity of the strategy was evaluated using cultivated MBVs strains, inactivated JEV vaccine strain, artificial VLPs, in vitro transcribed RNAs, and plasmids. The results showed that this assay genotyped MBVs and MBPs with no cross-reactions between the samples and reference materials (Fig. 3a, b). The CL signal value (i.e. that had not been calibrated) of each probe was shown in 3-D bar chart (Fig. 3c, d). Furthermore, a panel of non-MBVs did not yield any positive results with this strategy, suggesting the method is indeed highly specific (Additional file 1: Figure S1).

### Determination of LOD

**Fig. 4:**
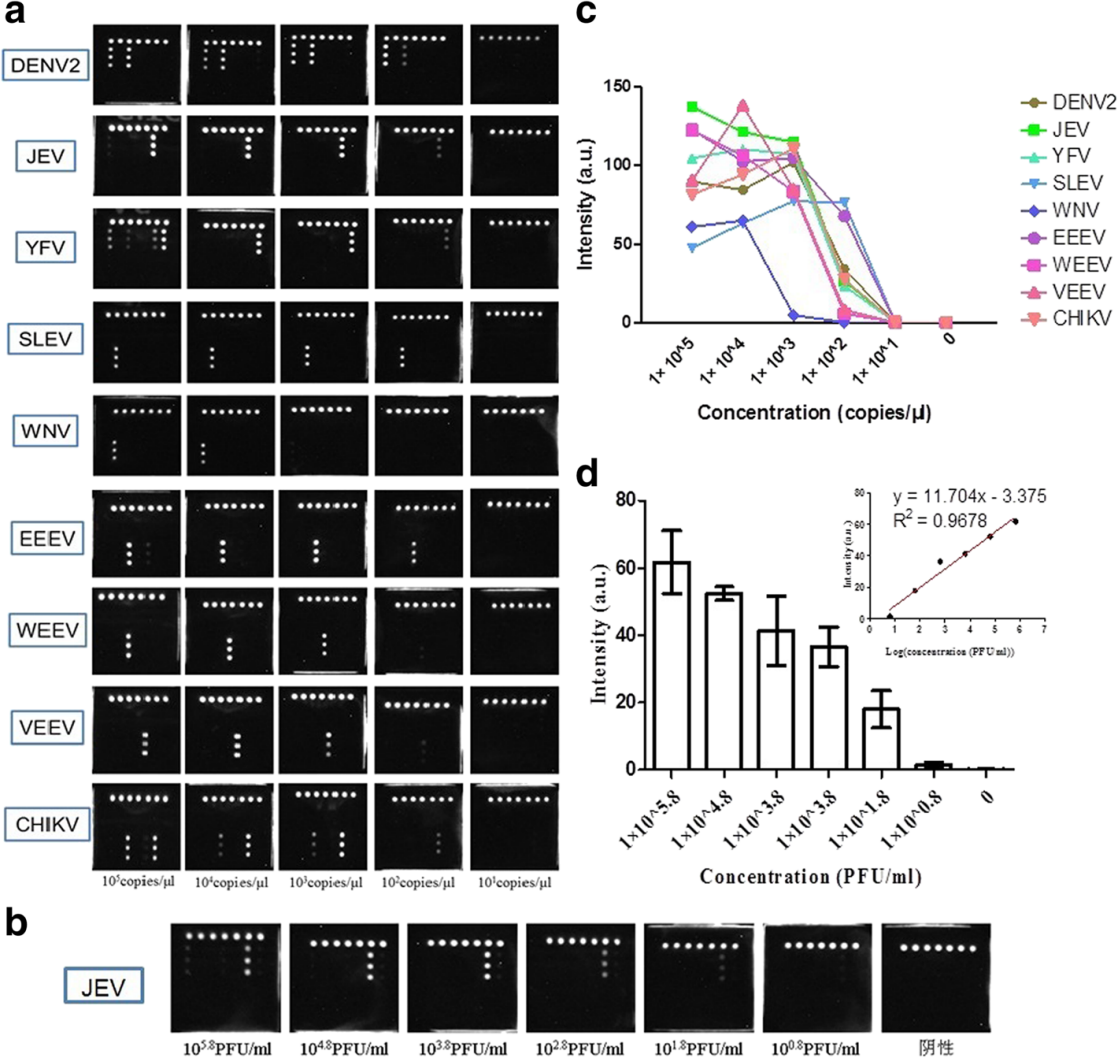
Determination of LOD for MBVs detection. **a** Detection of serially diluted in vitro transcribed RNAs of MBVs. The results show that the absolute LOD of this strategy to detect these MBVs is 10^2^ copies/μl except for WNV (10^3^ copies/μl). **b** Detection of a strain of JEV that had been accurately quantified by the National Institute for Viral Disease Control and Prevention. The LOD to detect JEV is between 10^1.8^–10^0.8^ PFU/ml. **c** Calibrated CL signals of diluted in vitro transcribed RNAs of MBVs. **d** Calibrated CL signal values are plotted as a linear function of JEV concentrations (inset). It fits well in a linear regression model. The coefficient of determination is 0.9678. Data shown are representative of two independent experiments

**Fig. 5:**
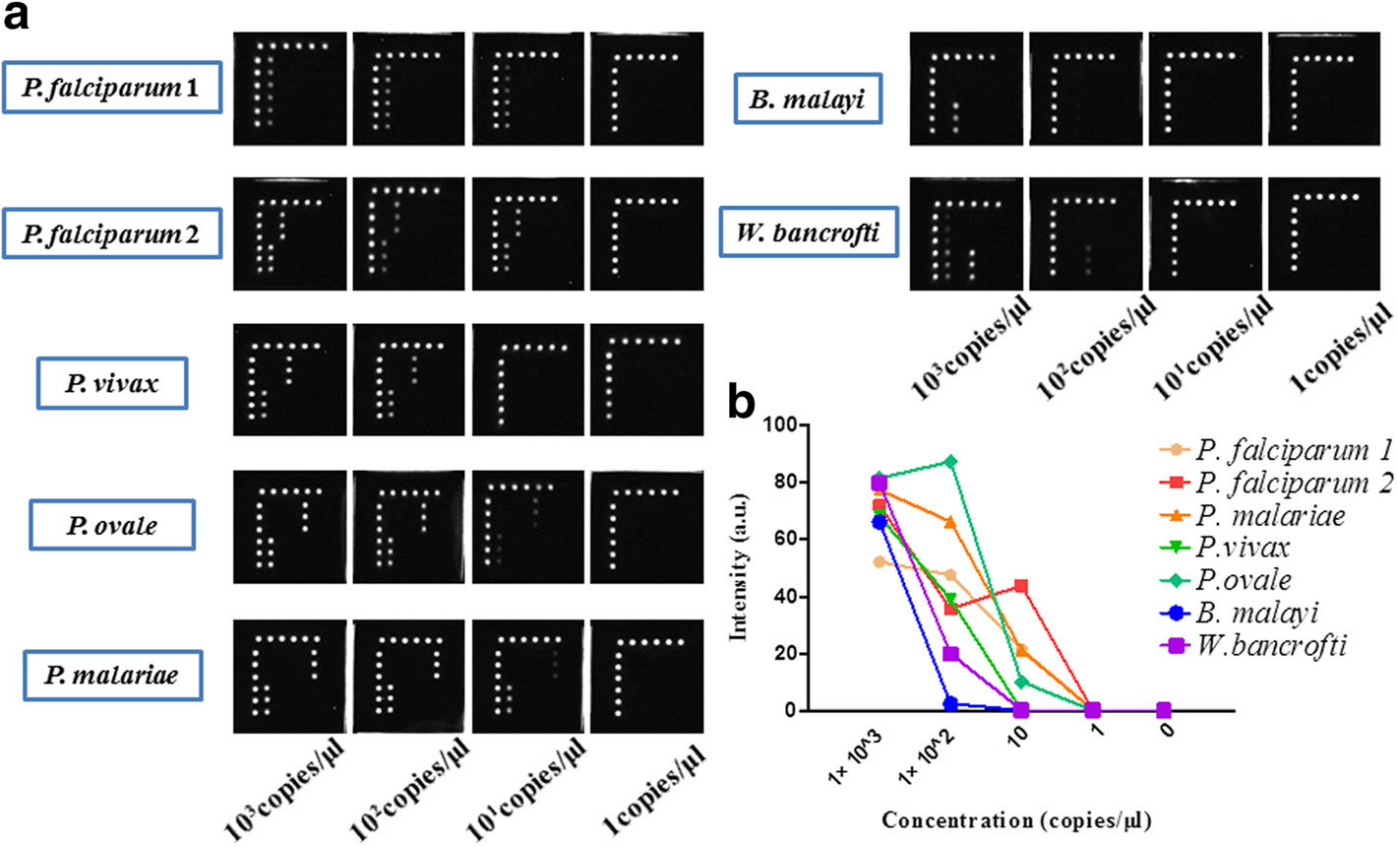
Determination of LOD for MBPs detection. **a** Absolute LODs for *P. falciparum*, *P. ovale*, and *P. malariae* is 10^1^ copies/μl and 10^2^ copies/μl for the remaining species. **b** Calibrated CL values of plasmid DNAs are shown in a line chart

To determine the absolute LOD of this strategy, serially diluted in vitro transcribed RNAs or plasmids, which had been quantified by the copy numbers, served as the reference materials. The absolute LODs for the MBVs were 10^2^ copies/μl except for WNV (10^3^ copies/μl), (Fig. 4a). The calibrated CL signal values of the in vitro transcribed RNAs are described in a line chart (Fig. 4c). Further, the absolute LODs for *P. falciparum*, *P. ovale*, and *P. malariae* are 10^1^ copies/μl; for the others, the values are 10^2^ copies/μl (Fig. 5a). The calibrated CL values of these plasmid DNAs are also described in a line chart (Fig. 5b)

A strain of JEV virus derived from National Institute for Viral Disease Control and Prevention was also employed as a reference material to determinate the LOD of this strategy. The results showed that the LOD for JEV was between 10^1.8^ PFU/ml and 10^0.8^ PFU/ml (Fig. 4b). A linear regression model based the calibrated CL signal values against the JEV concentrations shows satisfactory fitness, with the coefficient of determination being 0.9678 (Fig. 4d).

### Simulated detection of mosquito samples

**Fig. 6:**
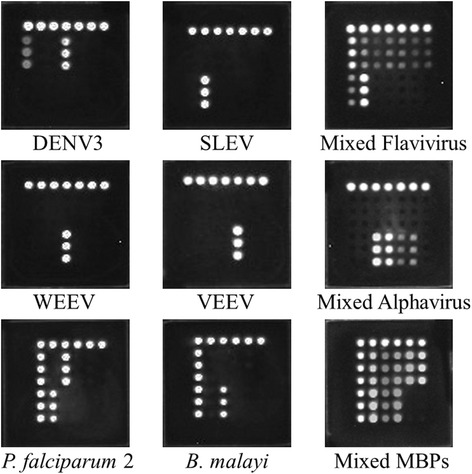
Simulated detection of mosquito samples. Uninfected mosquito pools of 50 *Aedes vigilax* were spiked with 10^6^ copies/μl of single VLPs (DENV3, SLEV, WEEV, or VEEV), single plasmids (*P. falciparum* or *B. malayi*), mixed VLPs of mosquito-borne flavivirus (including DENV1-4, JEV, YFV, SLEV, and WNV), mixed mosquito-borne alphavirus (including EEEV, WEEV, VEEV and CHIKV), or mixed plasmids of MBPs (including *P. falciparum*, *P. vivax*, *P. malariae*, *P. ovale*, *W. bancrofti* and *B. malayi*). This assay could detect single MBVs and MBPs in the mimic samples, as well as mixed mosquito-borne flavivirus, mixed mosquito-borne alphavirus, and mixed MBPs

To test simulated mosquito samples, uninfected mosquito pools of 50 *Aedes vigilax* were spiked with 10^6^ copies/μl of six single VLPs and plasmids or mixed VLPs and plasmids, followed by the CL imaging DNA hybridization assay. The results showed that this assay could detect all mimic samples of single MBVs, single MBPs, mixed mosquito-borne flavivirus, mixed mosquito-borne alphavirus, and mixed MBPs (Fig. 6). Therefore, the proposed assay can potentially test targeted MBVs and MBPs in actual mosquito samples.

## Discussion

As a competent vector, arthropods can support the sufficiently large inoculum of the virus and transmit the MBVs from an infected donor to a recipient host during blood-feeding. MBVs are also transmitted between male and female mosquitoes or from a female to the offspring. Furthermore, malaria and lymphatic filariasis, transmitted by hematophagous arthropods, are still the most prevalent mosquito-borne parasitic diseases in humans [13, 14]. A continuous effort to monitor the MBVs is critical to prevent sudden outbreaks [20]. Many gene-based diagnostic methods have been used to detect MBVs and MBPs. However, to the best of our knowledge, a portable, ultra-sensitive CL DNA hybridization method to simultaneously detect common MBVs and MBPs has not been reported. Because of the lack of antiviral drugs [10, 31, 32], the use of vaccines has emerged as an efficient strategy to control the spread of MBV diseases [33]. At present, human vaccines for flavivirus and alphavirus are available; however, these only include prevention of YFV, JEV, and TBEV [34–38], while the human vaccines for the prevention of DENV, WNV, CHIKV, SLEV, WEEV, EEEV and VEEV are still not approved for clinical trials [33, 39]. As a result, it has become imperative to develop reliable methods for rapid diagnosis, timely prevention, and efficient control of the mosquito-borne diseases. Rapid diagnosis is not only the indispensable method for controlling the infectious diseases, but also the prerequisite for timely prevention and efficient treatment. Infectious diseases are only able to be effectively prevented and treated after rapid and accurate diagnosis. Therefore, the spread of these infectious diseases, for which the drugs are not yet established, can be effectively reduced by controlling their vectors and accurate monitoring. In this assay, we developed a portable, ultra-sensitive CL imaging DNA hybridization method to detect various MBVs and MBPs simultaneously. This method is based on horseradish peroxidase catalyzing luminol-H_2_O_2_, and its performance proved to be highly sensitive and specific for the 12 MBVs and 7 MBPs that were included in the current study. The traditional epidemic of these pathogens involves many tropical and sub-tropical areas of Asia, Africa, the Americans, Europe and the Pacific archipelago [40–43]. Currently, there may not be a region in the world where all 19 pathogens are present. So the inclusion of the 19 pathogens in the current study appears exceeding the needs of clinical detections. However, we believe our study delivers a proof-of-concept study where such a diagnostic assay may test for more pathogens and save significant labour and time when a patient presents with symptoms of unknown etiology. Furthermore, given the fact that mosquito-borne diseases may occur in non-endemic areas due to the uncertainties of global human migration, for example, the first imported cases of Zika virus in China and Europe [44, 45], a rapid and ready-to-be-applied method will have significant clinical implications. For point of care testing, a self-powered portable CL CCD imager was developed. A self-programming software was employed to interpret the software readings and to study the grey values of the probes. However, other commercial CL imagers based on CCD imaging technology can also be used for CL imaging. Despite needing a CL imager, this CL imaging method has lower cost and faster detection speed than our previous visual method which was based on quantum dot-catalyzed silver deposition [30].

In the current study, due to the lack of pathogen nucleic acid samples, we validated the specificity of our method using the positive and negative references. The absolute LOD, which was evaluated using serially diluted in vitro transcribed RNAs and plasmids, was in the range of 10^1^–10^3^copies/μl. A strain of JEV was also employed as a reference material to determine the absolute LOD. Results from the two repeat tests showed that the absolute LOD for JEV was in the range of 10^0.8^–10^1.8^PFU/ml, and fits well in a linear regression model, demonstrating the feasibility of quantitative measurement of viral load. The assay is rapid, portable, ultra-sensitive, and high-throughput. The entire detection time, spanning from sample extraction to result reading, is between 6–8 h.

Since most of the targets in this assay are potent pathogens, we did not collect actual samples or all the nucleic acids. Instead, 14 artificial target pathogen gene sequences were constructed by overlap extension method and cloned into plasmid vectors as well as those target pathogens having nucleic acids samples (datas not shown). Subsequently, RNAs of these target viruses were in vitro transcribed from these plasmids. We observed that DNA templates from the plasmids were not completely removed by DNase I digestion. Consequently, RNeasy Mini Kit (QIAGEN), a purification method based on spin column, was also employed to purify and digest these in vitro transcribed RNAs on column. Although residual DNA templates were not completely removed from in vitro transcribed RNAs, they were negligible after the concentrations of in vitro transcribed RNAs were diluted to lower than 10^8^ copies/μl due to the twice DNase I digestion (dates not shown). Similar issue was observed during the preparation of VLPs of these target MBVs. However, to maintain the integrity, the VLPs were not digested by DNase I on the spin column. Consequently, the VLPs, as RNA positive reference materials, were also diluted to a sufficiently low concentration to overcome the influence of residual DNA templates as the concentration of residual DNAs were only 10^3^–10^4^-fold lower than that of the RNAs within VLPs.

In the present assay, a uniform reverse primer located in the 3′UTR region of flavivirus genome was employed in the multiplex asymmetric RT-PCR. Consequently, relatively shorter probes were used for flavivirus to eliminate cross signals among flavivirus species due to the homology of the genomic sequences of the 3′UTRs. On the other hand, our method did not distinguish between *B. malayi* and *B. timori* because the target sequences (i.e. HhaIrepeat sequence) are almost identical. Furthermore, methods based on DNA hybridization inevitably appeared cross signal with off target capture probes, especially for the samples containing extremely high concentrations of the target pathogens. Consequently, the cut-off values of capture probes showed in Table 5 need to be adjusted and refined to test the different concentrations of the target pathogen for future application. At present, there are many commercial extraction kits available to extract genomic RNA and DNA from diverse origins, so the proposed strategy can potentially detect these MBVs and MBPs regardless of the origins of these pathogens. In the proof-of-concept experiment, we spiked uninfected mosquito pools with VLPs or plasmids, demonstrated that our CL imaging DNA hybridization method could detect target MBVs and MBPs in mosquito samples. Therefore, further research should be focused on extending the method into the clinical settings for the epidemiological investigations of MBVs and MBPs.

## Conclusions

Ultra-sensitive CL imaging DNA hybridization was developed and could simultaneously detect MBVs and MBPs. This method is based on horseradish peroxidase catalyzing luminol-H_2_O_2_, and its performance proved to be rapid, portable, high-throughput, highly sensitive and specific. The entire operation time, spanning from sample extraction to result reading, is between 6–8 h. The method described here has the potential to provide considerable labor savings due to its ability to screen for 19 mosquito-borne pathogens simultaneously.

## Additional file

Additional file 1:
**Figure S1.** Determination of the specificity of the MBVs detection by detection of non-MBVs. A panel of non-MBVs were detected by this MBVs detection strategy to determine the specificity. There were no positive results (i.e. detection of these non-MBVs). YFV and EEEV were used as positive references. (XML 7 kb)

## Abbreviations

CHIKV: Chikungunya virus
CL: Chemiluminescence
DENV: Dengue virus
DNase: Deoxyribonucleas
EEEV: Eastern equine encephalitis virus
GSPs: Gene-specific primers
HBV: Hepatitis B virus
HCV: Hepatitis C virus
JEV: Japanese encephalitis virus
LOD: Limit of detection
MBPs: Mosquito-borne parasites
MBVs: Mosquito-borne viruses
QC: Quality control
RSV: Respiratory syncytial viruses
SDS: Sodium dodecyl sulfate
SLEV: St. Louis encephalitis virus
SSC: Saline-sodium citrate buffer
TBEV: Tick-borne encephalitis virus
VEEV: Venezuelan equine encephalitis virus
VLPs: Virus-like particles
WEEV: Western equine encephalitis virus
WHO: World Health Organization
WNV: West Nile virus
YFV: Yellow fever virus
